# A Corn‐Based Electrically Conductive Glue for Integration of Edible Electronics

**DOI:** 10.1002/smsc.202400373

**Published:** 2024-12-05

**Authors:** Noemí Contreras‐Pereda, Valerio Galli, Pietro Cataldi, Valerio Francesco Annese, Giulia Coco, Athanassia Athanassiou, Alessandro Luzio, Mario Caironi

**Affiliations:** ^1^ Center for Nano Science and Technology Istituto Italiano di Tecnologia Via R. Rubattino, 81 20134 Milan Italy; ^2^ Department of Physics Politecnico di Milano Piazza Leonardo da Vinci, 32 20133 Milan Italy; ^3^ Smart Materials Istituto Italiano di Tecnologia via Morego 30 16163 Genova Italy

**Keywords:** activated carbon, conductive adhesive, edible electronics, green electronics, zein

## Abstract

Edible electronics leverages the electronic properties of food‐grade materials to create non‐toxic technologies that can be either environmentally degraded or digested by the body after the completion of their function. Various edible electronic components have been recently proposed, and their integration into more complex circuits and systems is urgently needed for point‐of‐care devices. In this context, developing a safe technology for interconnecting edible components is crucial. To this aim, here an edible electrically conductive adhesive made from zein, an edible protein derived from corn, and activated carbon, a food additive, are reported. Different formulations are proposed depending on the ratio between adhesive binder (zein) and electrically conductive filler (activated carbon), evidencing a trade‐off between resistivity and adhesion, passing from a 3 × 10^3^ Ω cm resistivity and 2 MPa lap shear adhesion strength to 5 × 10^2^ Ω cm and 0.5 MPa values upon increasing the filler content. As a proof‐of‐concept, the conductive adhesive is validated in different applications relevant to edible electronics, such as mounting devices on top of innovative edible substrates, interconnecting state‐of‐the‐art edible batteries, and conforming highly adhesive electrodes for fruit monitoring.

## Graphical abstract

An electrically conductive glue is formulated using only food‐grade materials (zein from corn and activated carbon) for the development of complex circuits in the field of edible electronics. As a proof‐of‐concept, the edible glue is applied in different examples, such as device‐mounting on edible substrates, interconnection of edible batteries, and impedance analysis electrodes for fruit.

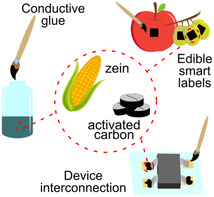

## 1 Introduction

Edible electronics is a research area focused on developing disposable functional devices in which all components, including powering devices, electronic circuits, sensors, actuators among others, are designed to be safely ingested and processed by the gastrointestinal system.^[^ ^1^ ^]^ Numerous high‐impact applications would result from electronic devices with complex functionality that, from a safety perspective, can be handled similarly to food. These applications include food‐quality monitoring tools to prevent food spoilage^[^ ^2^, ^3^ ^]^ and smart capsules for diagnosing and treating gastrointestinal disorders.^[^ ^4^, ^5^ ^]^ The swift evolution of the field has led to the development of distinct edible electronic components. Exemplarily, edible powering devices have been achieved like supercapacitors,^[^ ^6^, ^7^ ^]^ triboelectric nanogenerators,^[^ ^8^, ^9^ ^]^ and a first fully edible and rechargeable battery.^[^ ^10^ ^]^ Also, several edible sensors with strain,^[^ ^11^ ^]^ tilt,^[^ ^12^ ^]^ and pH^[^ ^13^ ^]^ sensitivity have been conceived, opening the door to multiple monitoring applications. Further, efforts toward developing edible circuits have provided electrolyte‐gated transistors fabricated on edible substrates as ethyl cellulose and using edible electrolytes as honey or chitosan.^[^ ^14^, ^15^ ^]^ Finally, silk‐based antennas for edible sensors^[^ ^16^ ^]^ have been developed for signal communication. A fundamental and still largely unexplored aspect of manufacturing edible electronic devices is identifying additive strategies for the assembling of separate components. A key challenge is finding alternatives to soldering and wiring widely used in traditional electronics, able to provide effective and stable electrical interconnections between all parts while being compliant with edibility constraints, including toxicity, size, and shape factors, and ensuring both electrical and mechanical efficiency. Given these premises, it is straightforward that the development of conductive adhesives made from edible ingredients and with a small footprint may represent a unique enabler for interconnecting edible components. Conductive adhesives are typically composed of a binding agent with adhesive properties and an electrically conductive filler, in sufficient quantities to establish efficient charge percolation through its bulk.^[^ ^17^ ^]^ Recently, it was demonstrated that gelatin‐based^[^ ^11^ ^]^ or beeswax‐based^[^ ^18^ ^]^ edible composites can achieve remarkable electrical conductivity through the incorporation of the right amount of activated carbon. Activated carbon is a highly porous form of carbon derived from vegetal sources, such as wood and coconut shells, which has been approved by international food agencies such as Food and Drug Administration and European Food Safety Authority for human consumption in virtue of its medical benefits to the gastrointestinal tract (food additive E153).^[^ ^19^ ^]^

Nevertheless, the reported composites are conceptualized as resistors or resistive sensors instead of adhesives. Several edible adhesives can be found in the literature as starch, gelatin, or sugar‐based glues, which have proven adhesion strength in the 100 kPa–MPa range.^[^ ^20^ ^]^ Interestingly, some of them, for instance starch, methyl‐ethyl cellulose (food additive E465), or polyvinyl alcohol (food additive E1203), have been employed as the binding agent in conductive adhesive formulations;^[^ ^21^, ^22^, ^23^ ^]^ nevertheless non‐edible conductive fillers were employed to provide charge percolation, such as soot or graphite. Among all, an effective binding agent largely employed in adhesive formulations is zein, a prolamine protein found in corn.^[^ ^24^, ^25^ ^]^ Zein typically exhibits adhesion strength of hundreds of kPa when applied with water‐based solutions,^[^ ^26^, ^27^ ^]^ which can be enhanced up to the MPa range when employed in resin formulations, that is, formulations including cross‐linkers such as tannic acid or other catechols that, upon high temperatures curing, promote superior film cohesion and interfacial adhesion.^[^ ^25^, ^28^ ^]^ The structure of zein is characterized by hydrophobic segments interconnected with hydrophilic glutamine‐rich chains.^[^ ^29^ ^]^ This enables zein to establish bonds with a wide variety of substrates: bonding either to hydrophobic substrates via van der Waals interactions between the substrate and the hydrophobic segments, or with hydrophilic substrates via hydrogen, ionic, or metallic bonds. Therefore, different adhesion forces for zein can be observed due to differences in the surface chemistry of the test substrates and thus the established bonds between the substrate and the zein.^[^ ^30^ ^]^ Zein‐based adhesives are particularly interesting in the context of edible electronics because they maintain their adhesion functionality even in water‐based environments, such as the body fluids of the gastrointestinal tract,^[^ ^31^ ^]^ and have already been used as binder in coatings for plant‐based electronics.^[^ ^32^ ^]^ In this work, we present an edible, electrically conductive adhesive ink, formulated using zein as the binding agent, activated carbon as the electrically conductive filler, and environmentally friendly solvents such as ethanol and water. The impact of activated‐carbon content on the rheology, adhesion strength, and electrical conductivity of the formulated ink was thoroughly investigated. The study revealed a 12‐fold enhancement in adhesive strength, reaching up to ≈2 MPa, when 20 wt% of activated carbon is used. This composition also resulted in a corresponding resistivity of ≈3 kΩ·cm, which is further reduced to ≈500 Ω·cm by increasing the activated‐carbon content to 30 wt%, at the expense of a loss in adhesion properties. Through the assessment of the prolonged electrical and adhesive durability of the material over several weeks, our edible conductive adhesive proved large reliability to effectively address the primary constraints of interconnecting edible electronics, as also evidenced by the illustration of exemplative practical applications. The connection of traditional surface‐mounted devices (SMD) on top of edible substrates and interconnection of edible batteries in series achieved with our adhesive pave the way to future novel edible logic systems and circuits for point‐of‐care devices; whilst conformal adhesive electrodes with our glue on top of fruit allow for non‐destructive and safe food impedance monitoring.

## 2 Results and Discussion

### 2.1 Formulation and Application of the Conductive Glue

**Figure 1:**
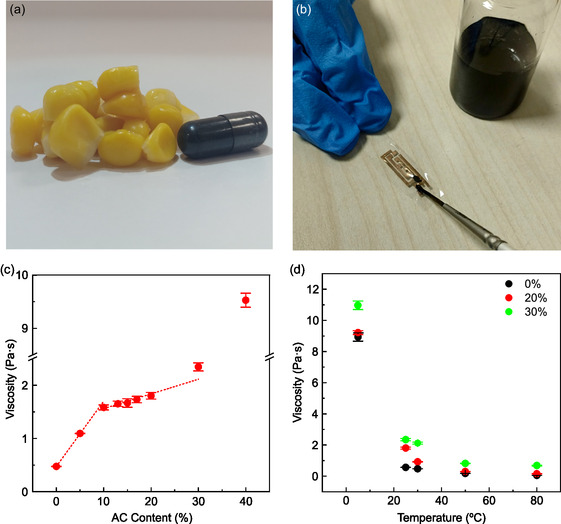
Composition, viscosity and painting of the conductive glue. a) The glue formulation is composed of zein corn protein and activated carbon (AC), food additive E153. b) Application of edible conductive glue by ink brushing for interconnection of printed gold electrodes on edible ethyl cellulose substrate. c) Average viscosity over three measurements for each edible glue as a function of the AC content. d) Average viscosity dependence with temperature over three measurements for each temperature for glues with 0, 20, and 30% AC content.

Edible conductive glues were formulated using zein from corn and activated carbon (AC, food additive E153, **Figure** **1**a). The zein and the AC powders were first mixed together by mechanical stirring to ensure their homogeneous blend, and then dissolved in a mix of ethanol and water (volume ratio 4:1),^[^ ^28^ ^]^ achieving a final zein concentration of 500 mg·mL^−1^. Different edible conductive glues were formulated by varying the AC content with respect to zein (Figure S1, Supporting Information), from 0% to 50% (wt%). For glues with 0% to 30% AC content, ink brushing at room temperature can be easily employed for local and small footprint “soldering‐like” applications as shown in Figure 1b, which are crucial in the microelectronic field, achieving acceptable reliability and control of the applied glue, mostly dependent on operator skill (Figure S2 and Videos S1–S3, Supporting Information).^[^ ^33^ ^]^ Ink brushing is also compatible with mask‐patterning techniques to achieve submillimetric resolution (Figure S3 and Video S4, Supporting Information). Upon drying, all brushed formulations present smooth solid surfaces, no longer sticky and firmly adhered to the substrate. Above 30% AC content, the formulated inks become incompatible with controlled ink brushing as large amounts of ink remain attached to the brush.

This small‐footprint applicability of the glues strongly depends on ink viscosity. As shown in Figure 1c and reported in Table S1, Supporting Information, the viscosity of the glue ink increases rapidly with the AC content from 0% to 10%, rising from 0.564 ± 0.002 to 1.583 ± 0.047 Pa·s, indicating good dispersion of the AC. At 10%, a discontinuity is observed in the trend, due to a reduced slope in the linear increase of viscosity with the AC content from 10% to 30%, where a moderate viscosity of 2.345 ± 0.076 Pa·s is measured. Then, in agreement with brushing deposition, a stronger discontinuity occurs at 40% AC content, with viscosity jumping to 9.528 ± 0.131 Pa·s. Finally, at 50% AC content, the glue starts behaving more like a solid resin than a fluid (see Video S5, Supporting Information), with a viscosity exceeding the measurement limit of the equipment; therefore, in the following we will focus on inks with AC content ≤30%. The temperature dependence of the viscosity for the 0, 20, and 30% AC content formulations are reported in Figure 1d. It can be observed that the viscosity of all inks exponentially decays with the temperature, in agreement with Arrhenius law (see Figure S4a, Supporting Information for fitting), with the 0% and 20% formulations displaying similar activation energies of 411 and 389 meV, while the 30% one showing a milder energy, of 217 meV. The latter indicates that up to 20% AC content, the temperature dependence of viscosity is dominated by zein, while for higher filler concentrations AC starts to affect the viscosity variation. Moreover, a reversible tuning of glue viscosity with temperature can be registered (see Figure S4, Supporting Information), highlighting the robustness of the glue formulation to thermal stress. This is important since, on the one hand, it makes possible the long‐term storage of the inks at low temperatures and its applicability after heating; on the other hand, the achievement of reduced viscosity approaching 10^−1^ Pa·s may open the way to a large variety of deposition techniques of technological interest, including large‐area coating, such as screen‐printing and blade/bar‐coating.^[^ ^34^ ^]^

### 2.2 Adhesion Strength of Edible Conductive Glues

**Figure 2:**
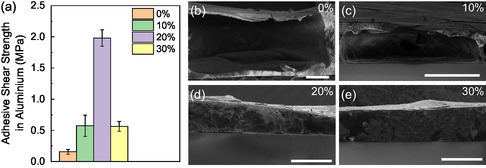
Morphology and adhesiveness of different formulations. a) Average adhesive shear strength on aluminum over three samples of the edible glues as a function of the composition. b–e) Cross‐section SEM images of brushed edible glues with different AC content after annealing at 80 °C. The AC content is indicated in each images. All scale bars are 200 μm.

The adhesion strength of the 10, 20, and 30% glues was evaluated and compared to pure zein (0%) to assess the impact of AC content on adhesiveness. Benchtop experiments already suggested a good strength of these edible conductive glues after drying (Figure S5 and Video S6, Supporting Information). Quantitative lap shear adhesion measurements using metallic aluminum substrates were performed to compare the obtained values to the ones reported in the literature for zein‐based glues. To this extent, the different glue formulations were deposited on top of the substrates by brushing, homogenously covering the area. Afterward, the substrates are pressed together and annealed for 2 h at 80 °C. We measured increasing mean adhesion strength values (**Figure** **2**a) of 0.16 ± 0.04, 0.57 ± 0.17, and 1.98 ± 0.13 MPa for 0, 10, and 20% formulations, respectively; the 30% AC content, glues conversely displays a drop of mean adhesion strength, down to 0.56 ± 0.08 MPa. All adhesion tests were highly reproducible, as evidenced by the low standard deviation in the adhesion strengths, which arises from the similar force–displacement curves reported in Figure S6a, Supporting Information. The values measured using pure zein (0%) are within the range of previous reports (200–700 kPa).^[^ ^26^ ^]^ Higher values are generally obtained by selectively choosing the protein formulation of the zein,^[^ ^35^ ^]^ or using cross‐linkers to reinforce the zein structure, such as tannic acid^[^ ^28^ ^]^ or cellulose nanofibers.^[^ ^36^ ^]^ Importantly, adding AC particles into the zein matrix leads to a neat improvement of the mean adhesion strength, up to 12× in case of 20% AC content, approaching 2 MPa. To our knowledge, lap shear strength values in the MPa range in zein‐based glues have only been achieved by using cross‐linkers requiring high annealing temperatures and long annealing times;^[^ ^25^ ^]^ conversely, we have obtained similar results with AC additives using a mild (80 °C) and short (2 h) thermal annealing, meant to allow the drying of the ink. We also confirmed the superior adhesion of AC‐containing zein inks by using glass substrates, which have substantially different surface chemistry with respect to aluminum and are also commonly employed in lap shear experiments (Figure S6b, Supporting Information). We measured adhesion strength values of 0.45 ± 0.16 MPa in the case of pure zein, while, in the case of 20% AC content, fracture of glass (Figure S6e, Supporting Information) occurs at 0.72 ± 0.06 MPa, before the detachment of the two substrates. The latter allows the assignment of adhesion force over 0.7 MPa to the 20% glue formulation. Furthermore, as shown in Figure S7, Supporting Information, the inclusion of the AC particles did not affect the adhesion properties of the zein underwater. As observed in pure zein adhesives,^[^ ^26^ ^]^ glued substrates maintained adhesion without spontaneous detachment after 24 h of immersion in water. The fracture of glass substrates evidences that substrate failure would occur if lap shear experiments were performed with edible substrates of interest for edible electronics, such as for example ethyl cellulose, on which quantification of adhesion force is not viable.

A deeper understanding of the zein inks adhesiveness mechanism can be obtained by analyzing the gluing areas after detachment upon lap shear stress. In fact, in lap shear experiments, two types of failure can be encountered: cohesive or adhesive failure. In the first, the adhesive strength is limited by a weaker internal cohesion and strength of the glue; therefore, the glue breaks internally, preserving the glued interfaces. In the second, an interfacial bond failure occurs between the adhesive and the glued substrates, as the glue strength is higher than the glue/substrate interfacial strength. Scanning electron microscopy (SEM) images of aluminum substrates after lap shear experiments proved a cohesive failure for the pure zein‐based edible glue, whilst glue containing AC displayed clear adhesive failure (Figure S8, Supporting Information). Therefore, we can likely address the improved adhesion of AC‐containing zein to an increase of the mechanical fracture toughness of the zein glue bulk, thus enhancing its overall adhesion force. This is consistent with SEM images of the cross‐sections of dried glue films with different AC content on glass (see Figure S9, Supporting Information for cross‐sections of all formulations). As seen in Figure 2b–e, 0% and 10% samples show large internal voids, making them more fragile. Upon increasing the AC content, the number and size of the voids decrease up to 20%, above which films appear compact. Therefore, we can expect that AC particles improve glue film compactness and, correspondently, their mechanical fracture toughness to lap shear stress. The 4× drop in the lap shear strength observed passing from 20% to 30% content may be ascribed to an excess of AC content, leading to just partial coverage of AC particles with the adhesive zein, as also suggested by SEM analysis in Figure S8, Supporting Information. Additional lap shear tests of the 20% and 30% samples, conducted on polyethylene naphthalene (PEN) substrates, confirmed the independence of such behavior on the substrate employed (Figure S6c, Supporting Information). Therefore, we can conclude that 20% AC content represents the optimal condition to maximize the adhesion properties of the edible conductive glues.

### 2.3 Electrical Performance of the Edible Conductive Glues

**Figure 3:**
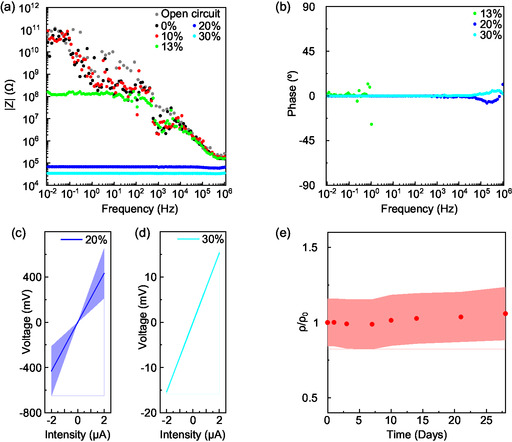
a) Modulus and b) phase of the electrical impedance spectroscopy measurements of the glues depending on the AC composition. c) Average four‐point I–V curve of 20% adhesive films over three samples. d) Average four‐point I–V curve of 30% adhesive films over three samples. e) Average four‐point resistivity measurement over three samples of the 20% edible conductive glue variation over time.

After assessing the adhesion strength, we performed the electrical characterization of formulations with variable AC content. First, we performed impedance analysis on formulations from 0% to 30% AC content, as reported in **Figure** **3**a (impedance modulus) and 3b (impedance phase; see Figure S10, Supporting Information for impedance analysis of all the formulations). All films were deposited using ink brushing and then annealed for 2 h at 80°C. 0% and 10% films behave as open circuits, therefore implying impedance modulus |*Z*| > 10^11^ Ω at 0.01 Hz. The minimum AC concentration needed to observe a resistive behavior, that is, the minimum quantity allowing charge percolation through the films, corresponds to 13% (Figure S10, Supporting Information), for which we observe 10^8^ > |*Z*| > 10^7^ Ω. From 20% on, a neat drop of |*Z*| is observed, down to the 10^4^–10^5^ Ω range, along with zero‐phase impedance, indicating the transition to an efficient charge percolation. For the same AC contents, a transition from porous to compact films was also highlighted (Figure S9, Supporting Information), suggesting a correlation between the structural change induced by the AC particles and the charge percolation efficiency within the films.

We performed four‐point measurements to extract the resistivity of ink‐brushed layers based on 20% and 30% formulations, that is, those formulations displaying efficient percolation (Figure 3c,d) and ohmic resistive behavior (Figure S11, Supporting Information). We have extracted mean resistivity values of 3 ± 1 kΩ·cm and 560 ± 170 Ω·cm for the 20% and 30% formulations, respectively. Such resistivities are in line with typical resistivity values of activated‐carbon‐based materials.^[^ ^11^, ^18^ ^]^ It is worth highlighting that the 5× increase of conductivity in the 30% sample comes at the expenses of a 4× drop in adhesion strength, representing a clear trade‐off between electrical and adhesion characteristics. Finally, we found stable average resistivity over one month in ambient storage conditions, as illustrated in Figure 3e.

## 3 Discussion and Outlook

The development of complex circuits and integrated systems in the edible electronics research field is strongly limited by the lack of consistent strategies to stably and electrically interconnect device components while complying with edibility constraints. In this work we have developed an edible conductive glue as a novel tool to interconnect edible electronic components. The conductive adhesive is made from food‐grade materials, such as zein (a protein from corn) and activated carbon (AC, food additive E153). The formulated inks, based on ethanol and water green solvents, were demonstrated to be compatible with standard local application techniques, such as ink brushing, largely employed with commercial glues, and with printing techniques such as blade coating (Figure S12, Supporting Information). We found that the inclusion of the electrically conductive activated carbon into the zein adhesive confers to zein outstanding mechanical robustness, with improved cohesive forces, and thus enhanced adhesiveness approaching 2 MPa, so far only achieved through cross‐linking and high‐temperature curing strategies. A minimum AC loading of 20% is necessary to provide efficient charge percolation into the glue, providing a resistivity of 3 × 10^3^ Ω·cm, which is further decreased to 5 × 10^2^ Ω·cm upon increasing the AC content up to 30%. Nonetheless, a trade‐off between adhesion and electrical conductivity enhancement sets 20% AC loading as a reasonable compromise privileging the superior and stable gluing character, preserving the chance to obtain an efficient and stable ohmic electrical junction between the glued surfaces (Figure S13, Supporting Information).

**Figure 4:**
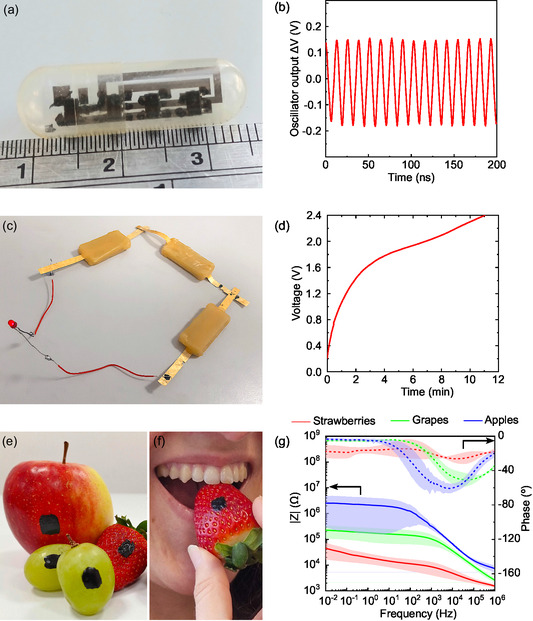
a) Photograph of ring‐oscillator made of three commercial CMOS inverters with an output buffer inverter mounted onto edible printed contacts with edible glue and fitted into a 00 commercial gelatin capsule. b) Voltage at the buffer output when providing 1.2 V. c) Photograph of three edible batteries in series powering a LED connected with the edible conductive glue. d) Charging curve at 300 μA of three edible batteries connected in series. e) Adhesive edible electrodes on top of an apple, grapes, and a strawberry made with our glue. f) The edibility of the deposited glue electrodes ensures its consumption along the fruit without toxicity issues. g) Mean absolute bio‐impedance modulus (full line) and phase (dashed line) over three strawberries, three grapes, and three apples measured after deposition of the glue as edible conductive electrodes.

In **Figure** **4**, we have collected three relevant proof‐of‐principle use cases for the edible glue. In Figure 4a, the assembly of commercial SMD inverters (Texas Instruments CMOS inverter SN74AUC1G04‐DBVR) to realize integrated ring oscillators onto an edible ethyl cellulose substrate^[^ ^1^, ^37^ ^]^ (E 462) is illustrated (a schematic of the circuit can be found in Figure S14, Supporting Information). The interconnecting conductive pattern was first pre‐printed on top of the ethyl cellulose substrate based on a CAD‐defined ring‐shaped circuital design, using Au inks (being gold a food additive E175) and ink‐jet printed technology.^[^ ^15^ ^]^ Successively, an odd number of inverters (3) were accurately positioned onto the pre‐printed edible circuit, and their pins were finely connected to the circuital conductive lines using the edible glue. The assembling achieved with our glue did not alter the flexibility of the edible substrate, which could be bent to fit the final circuit into commercial gelatin 00 capsules, as exemplarily needed for ingestible electronics. Additionally, the conductive pattern printed on ethyl cellulose interfaced with the glue does not delaminate after 6 months of application of the glue, nor over mechanical stress like manipulation, which highlights the good adhesion of our glue to ethyl cellulose and printed gold lines. As shown in Figure 4b, a stable and reproducible oscillation with a frequency of 78 MHz is observed when supplying a 1.2 V DC voltage, which is within recommended voltage supply range for this component (0.8–2.7 V). This evidence anticipates the possibility of finely assembling fully edible logic circuits upon mounting together future edible electrical components. In Figure 4c, three recently designed and reported edible rechargeable batteries^[^ ^10^ ^]^ were connected in series, to allow their charging and the powering of a red LED (HLMPK150 from RS Components), connected as well with our edible conductive ink. Millimeter‐sized interconnecting areas were coated via ink brushing with glue and pressed together; 30 min after the application, our edible conductive glue was dried, and the batteries were mechanically attached and electrically connected. Batteries were charged to 2.4 V after being connected in series, demonstrating that our edible conductive glue provided interconnection between them (Figure 4d). After being charged, the batteries were able to power the red LED, as shown in Figure 4c. The circuit showed time functioning of 15 min, which suggests a negligible resistive loss and large time stability of the glue as evidenced by the low resistance of the vertical junction between ethyl cellulose electrodes (Figure S15, Supporting Information).

In Figure 4e,f, the conductive ink is employed to realize non‐invasive and food‐grade electrical contacts for monitoring fruit bio‐impedance signals. Electrical impedance spectroscopy analysis is a widely known technique to monitor fruit quality and rotting, but is currently limited by the need of food‐safe electrodes with constant adhesive and electrical interface with fruits.^[^ ^38^ ^]^ Interestingly, the glue on the fruit's skin displays long‐term adhesion and highly conformal contact throughout the whole fruit lifetime (see Figure S16, Supporting Information), paving the way to fruit monitoring along ripening by electrical impedance analyses. Electrical bio‐impedance spectroscopy measurements were hence performed for all fruits, as shown in Figure 4g. Noteworthy, the measured module and phase of strawberries highly resemble measurements previously performed in the literature, validating the electrical contact obtained with our edible glue electrodes.^[^ ^39^ ^]^ We believe that the capacity of our edible conductive glue to mount complex circuits and interconnect devices can be of high interest to achieve novel logic systems in edible electronics. Finally, the large conformability of our glue toward complex surfaces as fruits anticipates its use for smart food labels, conferring edible long‐lasting electrodes for bio‐impedance monitoring of several different fruits and other perishable food.

## 4 Experimental Section

4.1

4.1.1

##### Materials

Zein with purity over 98% and puriss. p.a. Activated carbon (AC) (E153) (purity above 98.5%) were used as purchased from Sigma Aldrich. The purchased zein powder is a mixture of the four classes of zein (α, β, γ, and δ) found in corn. AC particle size is in the micrometer range with a graphitic like morphology. Absolute ethanol was purchased from Carlo Erba. Water was filtered, deionized, and decalcified.

##### Preparation of the Edible Glues and Inks

Zein (10 g) and AC powders (desired weight ratio to zein) were mixed by mechanical stirring until they were homogenously mixed to ensure a final good dispersion of AC within the zein matrix. Afterwards, 20 mL of 4:1 ethanol:water (volume ratio) solvent was added to attain a 500 mg mL^−1^ concentration of zein. This concentration is slightly lower than those used in zein‐based adhesives to obtain clear zein solutions,^[^ ^25^, ^31^ ^]^ further contributing to the good dispersion of AC. Mechanical stirring with a spatula was used as for commercial acrylic inks, until homogenous and lump‐free inks were obtained. High purity materials and identical mixing procedures were applied for all inks, ensuring only the AC content varies from one ink to the other. The inks were deposited by ink brushing technique with brushes for acrylic inks purchased in a local supermarket.

##### Blade‐Coating

Glass substrates were previously cleaned by sonication in acetone for 10 min, followed by sonication in isopropanol for 10 min, and dried with nitrogen. Blade coating was performed with TQC Sheen Automatic Film Applicator AB4400. The 20% glue was bladed at a 5 mm s^−1^ rate on top of glass substrates at different temperatures (room temperature, 30 °C, 50 °C, and 80 °C) and sub‐sequentially annealed at 80 °C with the blade at 0.5 mm of the substrate.

##### Rheology Measurements

The viscosity of glue inks was determined using a Fungilab Alpha series Rotational Viscometer with a L2 spindle for glues from 0% to 30% percent at room temperature and L3 spindle for the 40% glue. For temperature dependence, L2 spindle was used. Rotation speed was chosen accordingly to have a reading value. All viscosities were measured three times on the same ink. For viscosity variations with humidity, the measured ink was introduced in a Memmert HPP110 environmental chamber. Relative humidity was controlled between 60% and 80% at each temperature (room temperature, 30, 50, and 70 °C). The ink was kept for one hour at each condition of temperature and humidity, afterwards it was removed from the environmental chamber and three viscosity measurements were performed immediately after and averaged.

##### Lap Shear Adhesion

The lap shear strength was assessed utilizing a dual‐column Instron 3365 universal testing machine, equipped with a 2 kN cell load and screw‐action clamps, at a strain rate of 5 mm min^−1^. Measurements were conducted following the ISO 4587:2003 procedure. Initially, aluminum sheets measuring 100 × 25 × 1 mm and glass substrates measuring 75 × 25 × 1 mm underwent polishing using sandpaper. Subsequently, the aluminum sheets were cleansed sequentially with soap, distilled water, and isopropanol and the glass substrates were cleansed with distilled water and isopropanol. On the contrary, PEN substrates measuring 75 × 25 × 0.125 mm were cut and cleaned with distilled water and isopropanol without further treatment. Each conductive glue was then applied to the substrates through ink brushing, ensuring homogeneous coverage of the areas of ≈12 × 25 mm. Following this, all substrates were pressed together with identical clamps, covering one brushed area with another. Afterwards, all substrates were subjected to the same annealing for 2 h at 80 °C to avoid differences between glues in their drying. Each pair of glue and substrate underwen*t* testing a minimum of three times.

##### Scanning Electron Microscope

The morphology and thickness of the brushed and bladed materials were investigated by high‐resolution SEM using a TESCAN MIRA3 operating at an acceleration voltage of 5 kV. Brushed samples were prepared by ink brushing areas of 1 × 1 cm on top of glass for each edible conductive glue. Cross‐section images were obtained by previously cutting the substrate with a diamond tip and tilting the sample by 90°. The average thickness of the brushed inks after annealing was extracted from these images.

##### Electrochemical Impedance Spectroscopy

To measure the impedance of the conductive glues, samples were prepared as previously, brushing areas of 1 × 1 cm. Soldering cables were pasted on opposite sides of each sample with the same glue as the sample. Samples were dried for 2 h at 80 °C. Electrochemical impedance spectroscopy was recorded using a MultiPalmSens4 potentiostat by applying a sinusoidal stimulus of 100 mV within a frequency range of 10 mHz−1 MHz.

##### Electrical Characterization

To measure the sheet resistance, edible glues were deposited on top of glass substrates covering areas of 2.5 × 1.5 cm via ink brushing and dried for 2 h at 80 °C. Three identical samples were produced for each formulation (i.e., each AC loading condition). For blade‐coated samples, long‐bladed films were cut into three identical pieces of 2.5 × 1.5 cm cutting the glass substrate with a diamond tip. The sheet resistance was measured using a four‐probe configuration to eliminate the contribution from the contact resistance. Identical electrodes of *W* = 15 × 1 mm spaced at a constant distance of *L* = 6 mm were deposited on top of the dried glues (RS PRO conductive paint 186‐3600). The two external electrodes were connected respectively to ground and a sweeping current source using a B1500A Keysight Semiconductor Parameter Analyzer. The sheet resistance was then obtained by reading the voltage difference between the two internal electrodes, dividing it by the supplied current and multiplying for the geometrical factor W/L. The height of the coating was measured by imaging the section using a lateral microscope. The resistivity was then obtained by multiplying the sheet resistance with the coating height.

##### Ring Oscillator Mounting and Characterization

Edible ethyl cellulose substrates were obtained as previously reported.^[^ ^15^ ^]^ The CAD‐designed gold pattern was ink‐jet printed on top with a Fujifilm Dimatix DMP‐2831 printer with a Samba cartridge. The substrate was maintained at 60 °C during the printing process, and the electrodes were subsequently sintered at 120 °C in air for 20 min. Commercial Texas Instruments CMOS inverters SN74AUC1G04‐DBVR were purchased from RS Components (UK). The inverters were surface mounted onto the printed circuits via the edible conductive glue applied with millimeter‐wide brushes. Continuous DC supply voltage was applied to the ring‐oscillator circuit with a Keysight B2900A Series Precision Source while the oscillator's output voltage was read with a Tektronic MSO4000 series oscilloscope. Gelatin 00 capsules were purchased from Nadiprana S.L. (Spain).

##### Edible Rechargeable Batteries Fabrication, Interconnection, and Testing

Three edible rechargeable batteries were fabricated as described in.^[^ ^10^ ^]^ After fabrication, they were connected in series, gluing battery electrodes with edible conductive glue. The battery pack was then charged to 2.4 V with a charging current of 300 μA, using a MultiPalmSens4 potentiostat.

##### Fruit Electric Impedance Spectroscopy

Red apples, strawberries, and green grapes were purchased at a local supermarket. After gentle cleaning with water and soap to remove possible protective resins, two 1 × 1 cm square electrodes were applied via ink brushing our edible conductive glue on each fruit, on the equatorial region of each fruit, with one electrode diametrically opposed to the other to ensure the measurement across the fruit's pulp. Only one brush was applied per electrode to minimize the variation of electrode thickness. After electrode drying, one soldering cable was glued to each dried electrode with a single droplet of edible conductive and crocodile clips onto the cables were utilized for proper connection with the electrodes. Afterward, electrochemical impedance spectroscopy was recorded using a MultiPalmSens4 potentiostat by applying a sinusoidal stimulus of 100 mV within a frequency range of 10 mHz−1 MHz. Triplicates were run for each fruit.

## Conflict of Interest

The authors declare no conflict of interest.

## Author Contributions

**Noemí Contreras‐Pereda**: conceptualization (equal); data curation (lead); formal analysis (lead); investigation (lead); methodology (equal); validation (equal); writing—original draft (lead); writing—review and editing (equal). **Valerio Galli**: investigation (supporting); methodology (equal); validation (equal); writing—review and editing (supporting). **Pietro Cataldi**: investigation (supporting); methodology (equal); validation (supporting); writing—review and editing (supporting). **Valerio Francesco Annese**: investigation (supporting); methodology (supporting); validation (supporting); writing—review and editing (supporting). **Giulia Coco**: investigation (supporting); methodology (supporting); validation (supporting); writing—review and editing (supporting). **Athanassia Athanassiou**: methodology (supporting); supervision (supporting); writing—review and editing (supporting). **Alessandro Luzio**: conceptualization (supporting); investigation (supporting); methodology (supporting); supervision (equal); writing—original draft (supporting); writing—review and editing (supporting). **Mario Caironi**: conceptualization (equal); funding acquisition (lead); investigation (supporting); methodology (equal); project administration (lead); resources (lead); supervision (lead); validation (equal); writing—original draft (supporting); writing—review and editing (lead).

## Supporting information

Supplementary Material

## Data Availability

The data that support the findings of this study are available from the corresponding author upon reasonable request.
