# Supplementary material for: A Corn‐Based Electrically Conductive Glue for Integration of Edible Electronics
Source: Small Sci. 2024 Dec 5;5(1):2400373. doi: 10.1002/smsc.202400373 (PMC11935078; doi:10.1002/smsc.202400373)
Supplement: Supplementary file 1 — Supplementary Material [file SMSC-5-2400373-s001.zip › SMSC.202400373/SMSC.202400373-sup-0001-SuppData-S1.pdf]

Supporting Information

A corn-based electrically conductive glue for integration of edible electronics

Noemí Contreras-Pereda[a], Valerio Galli[a,b], Pietro Cataldi[c], Valerio Francesco Annese[a], Giulia Coco[a,b], Athanassia Athanassiou[c] Alessandro Luzio[a], Mario Caironi[a]**

[a] Center for Nano Science and Technology @PoliMi, Istituto Italiano di Tecnologia, Via R. Rubattino, 81, Milan, 20134 Italy

[b] Department of Physics, Politecnico di Milano, Piazza Leonardo da Vinci, 32, Milan, 20133, Italy

[c] Smart Materials, Istituto Italiano di Tecnologia, via Morego 30, Genova, 16163 Italy

1. Ink for glue manufacture and processing

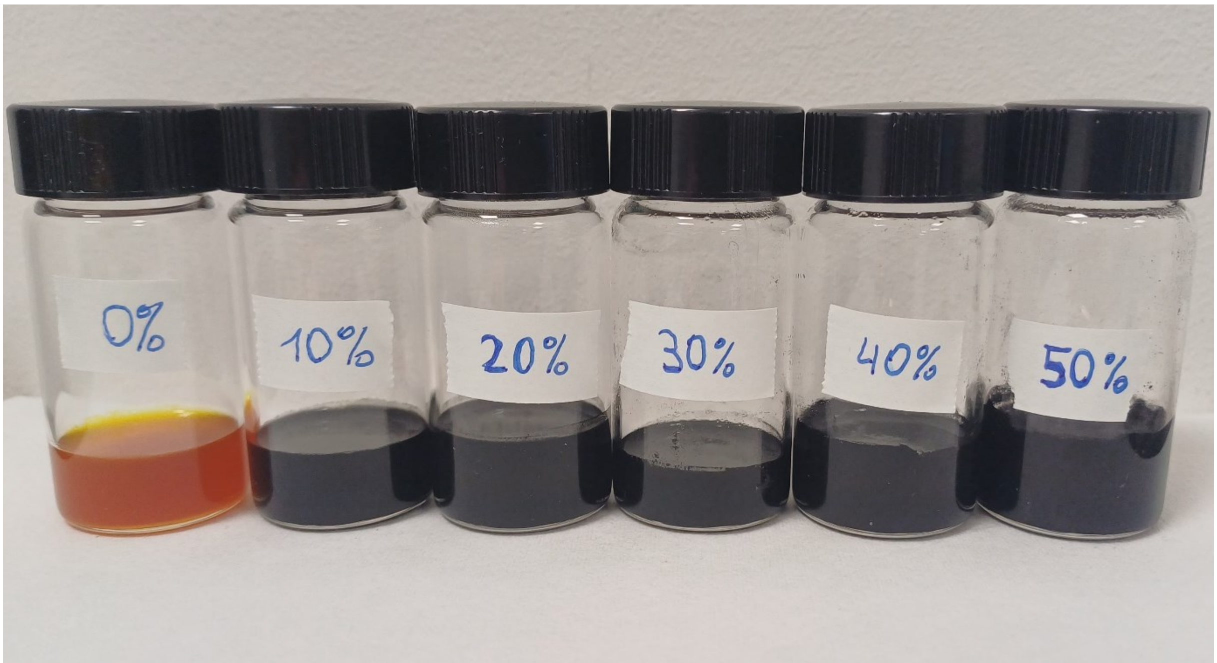

Figure S1. Photograph of different glue formulations depending on the AC content.

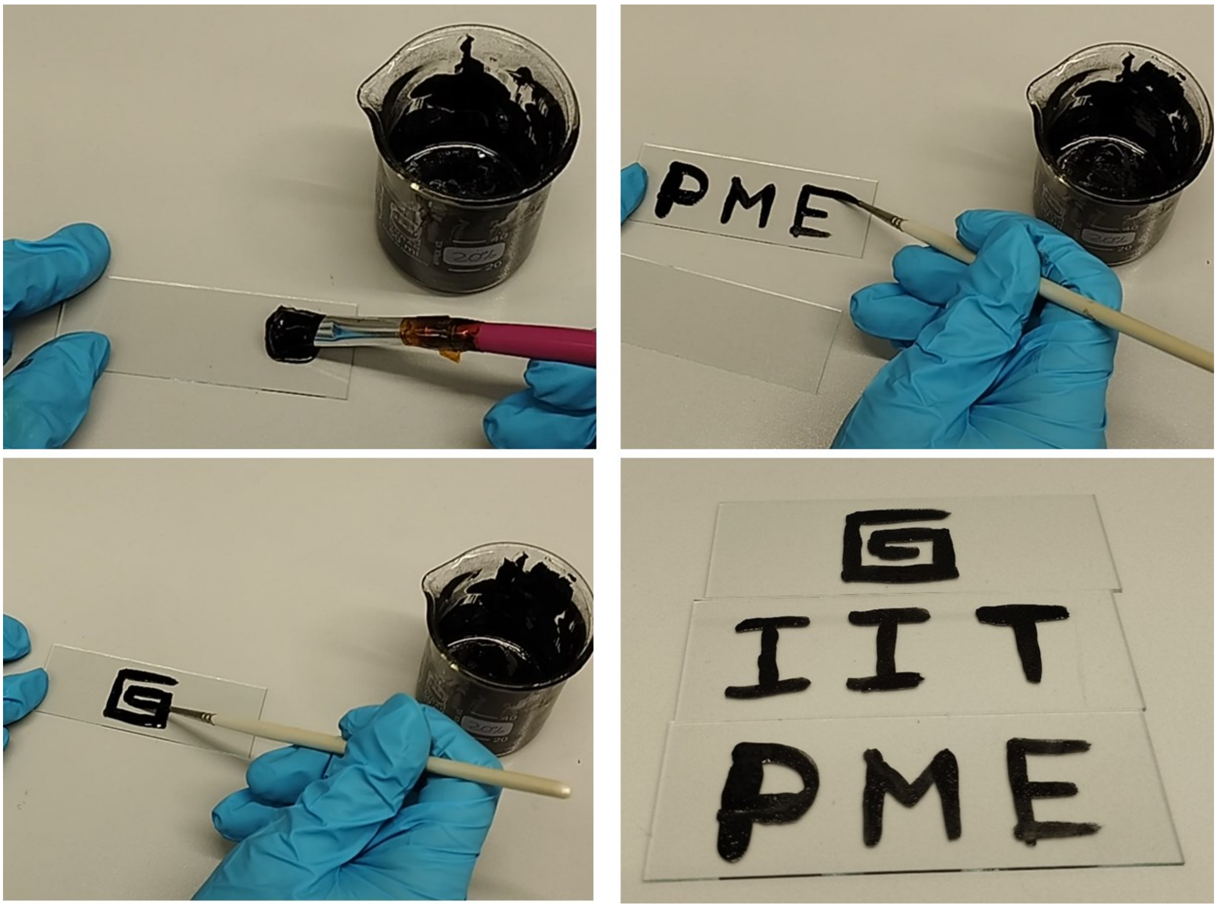

Figure S2. Photographs extract from videos of deposition via ink brushing.

The formulated edible glue inks can be applied using ink brushing techniques like those employed with commercial conductive adhesives in the field of microelectronics, utilizing a range of brush types (Figure S2). The deposition process is easily controlled based on the operator's skill, the amount of ink used, the pressure exerted with the brush, and the brush size. Consequently, this technique facilitates customized depositions, offering adaptability for constructing a diverse range of circuits.

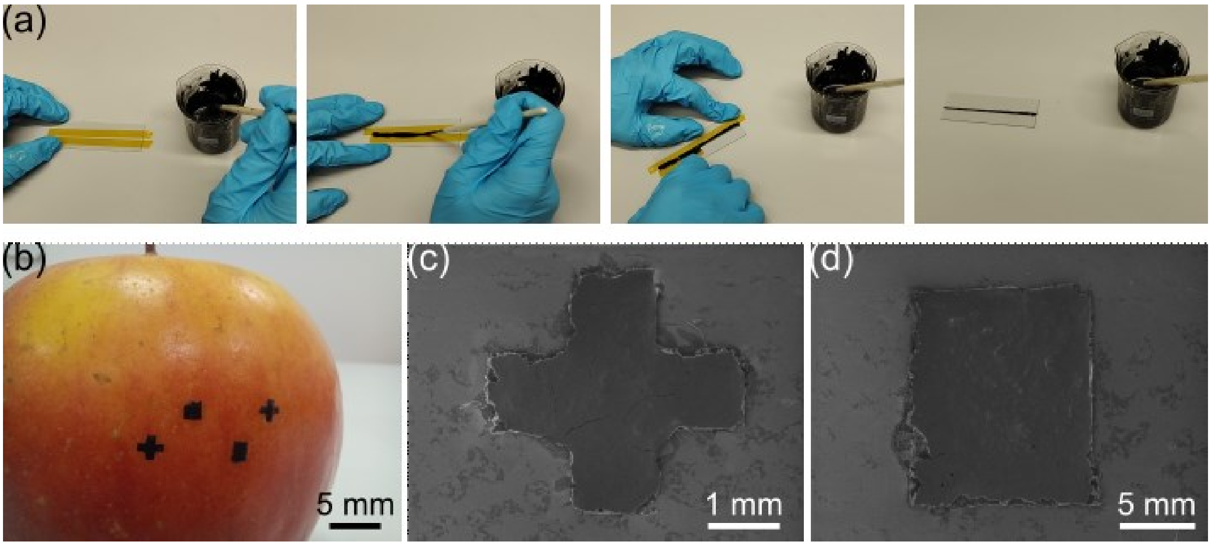

Figure S3. (a) Photographs extract from video showing the compatibility of our edible conductive inks with tape masking to have thin depositions. (b) Photograph of tailored shapes deposited by tape masking on top of an apple. (c) and (d) SEM images of tailored shapes obtained with masking on top of glass substrates.

Ink brushing deposition is compatible with mask-patterning techniques to achieve micrometric resolution (Figure S3). Well-defined shapes could be attained through mask patterning using tape. Thin lines were created by brushing ink between tapes, which were subsequently removed after the ink dried, as illustrated in Figure S3a. More intricate or customized shapes can be achieved by blade-cutting the tape, allowing the deposition on either glass or complex surfaces as on apple skin (Figure S3b). As seen in Figures S3c and S3d, submillimeter resolution can be achieved with masking resulting in continuous and compact depositions.

Table S1. Viscosity values of the different glue formulations depending on the AC content.

Edible glue (% AC)	Viscosity (Pa·s)
0%	0.564 ± 0.002
5%	1.094 ± 0.006
10%	1.583 ± 0.047
13%	1.652 ± 0.049
15%	1.666 ± 0.080
17%	1.732 ± 0.062
20%	1.803 ± 0.064
30%	2.345 ± 0.076
40%	9.528 ± 0.131

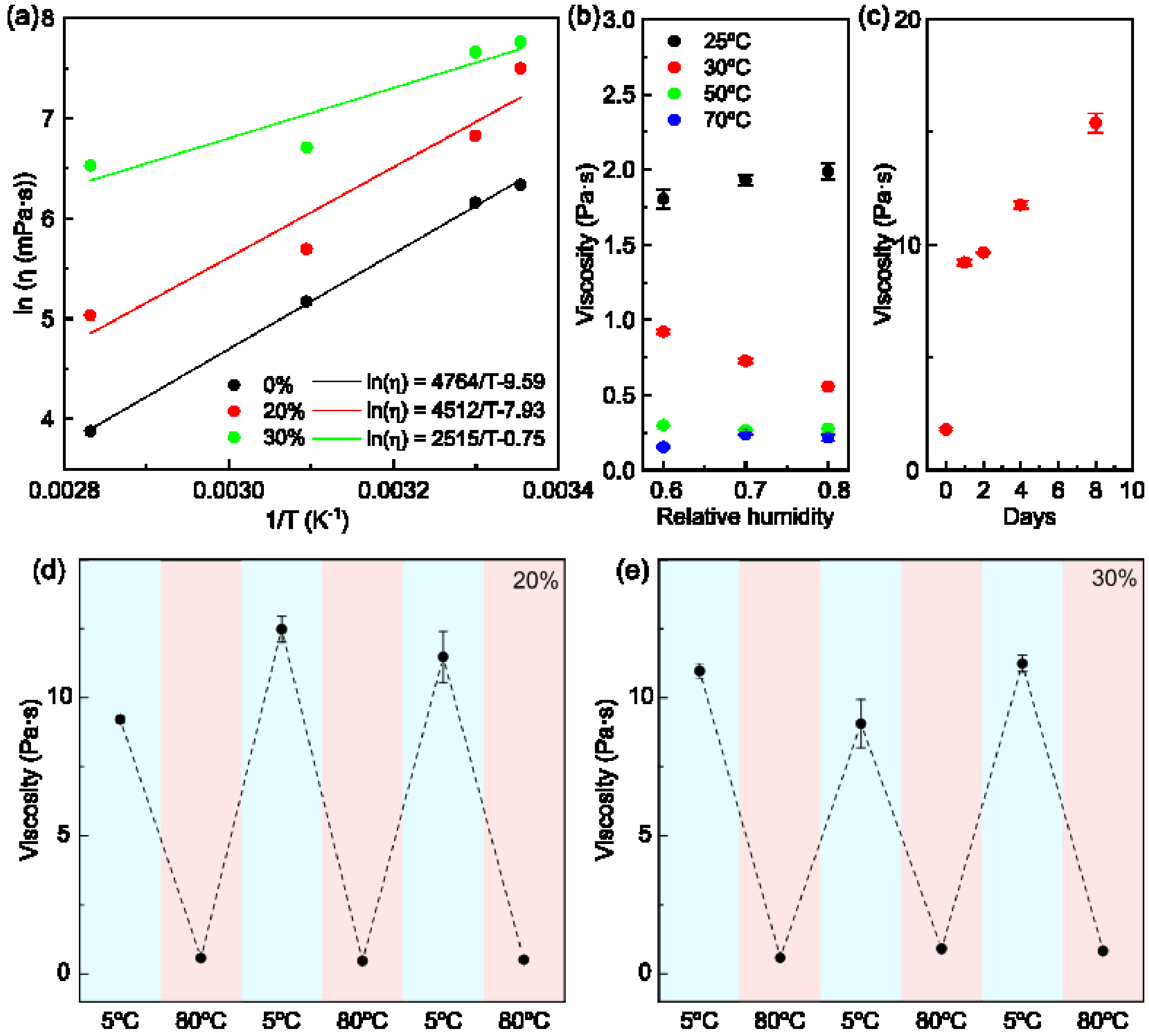

Figure S4. (a) Viscosity variation of the 0%, 20% and 30% glue inks with temperature fitted in an Arrhenius dependence. (b) Viscosity of 20% edible glue at different temperatures and humidity. (c) Viscosity along time of the 20% edible conductive glue at 5ºC. Viscosity of the (d) 20% and (e) 30% glue inks after cycles of storage at 5ºC for 24h and reheating at 80ºC.

Viscosity dependence to ambient factors was studied since it can affect the small-footprint applicability. Viscosity variation with temperature was fitted to an Arrhenius equation as shown in Figure S2a in a 20-80ºC range since the 5ºC data point was not following the Arrhenius law for any of the temperatures. The equation is read as the following:

$$\ln(\eta) = \ln(A) + \frac{E_A}{R}\frac{1}{T}$$

Where η is the viscosity, T the temperature, R the constant of gases and A and E_A are characteristic values of the ink, namely, the preexponential entropic factor and the Arrhenius activation energy.[1] Taking into account R = 8.63×10^{-5} eV·K^{-1}·atom^{-1}, activation energies of 411; 389 and 217 meV are extracted for 0%, 20% and 30% inks respectively. Viscosity variation of the pure zein ink and the 20% AC present similar preexponential factor and activation energy than the 0%. Therefore, the variation of viscosity with temperature in the 20% seems to be governed with the zein. On the other

hand, a lower activation energy is registered for the 30% glue, suggesting AC is participating to viscosity modification along with zein.

Effect of the relative humidity at different temperatures was also studied in Figure S2b since water has been proven to be a plasticizer for zein and therefore might affect viscosity.[2] Due to equipment limitations, variations with humidity were studied at 70ºC instead of 80ºC. At 25 ºC, close to room temperature (RT) conditions, a slight viscosity increase is obtained with increased humidity whilst at 30 ºC, an opposite behavior with RH is observed since viscosity decreases with increasing humidity. Similar viscosity values are achieved at 50 ºC independently of the RH suggesting a limit in low viscosity is being attained. Indeed, upon increasing the temperature to 70 ºC the slightly lower viscosities ranging from 154 to 240 mPa·s at different RHs are achieved. Hence, relative humidity seems to affect the viscosity of the inks only at low temperatures.

Storage of the inks at ambient temperature results in the drying and hardening of the glue even when stored in sealed recipients. Therefore, the viscosity along time in a closed recipient at a 5 ºC ambiance was assessed to study possible storing at low temperatures as done with other commercial glues (see Figure S2c). Remarkably, the viscosity increases drastically upon storing it at cool temperatures, reaching 57.4 ± 2.3 Pa·s after 14 days of storage. Noteworthy, even after two weeks of storage of the glue in the fridge, viscosities of 0.582 ± 0.003 Pa·s can be easily recovered upon heating them up at 80 ºC. These values are highly similar to the ones of fresh samples at high temperatures. This tuning of glue viscosity with temperature has proven to be reversible (see Figure S2e-f), suggesting limited ink degradation due to thermal stress. Therefore, prolonged storing and following small footprint is enabled.

2. Adhesion strength of edible glues

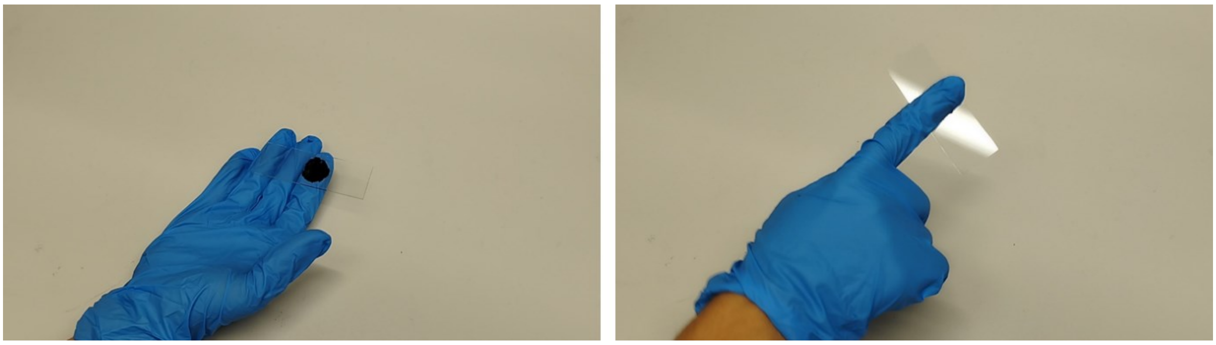

Figure S5. Photographs extracted from video showing good adhesion of the edible glues of common lab objects.

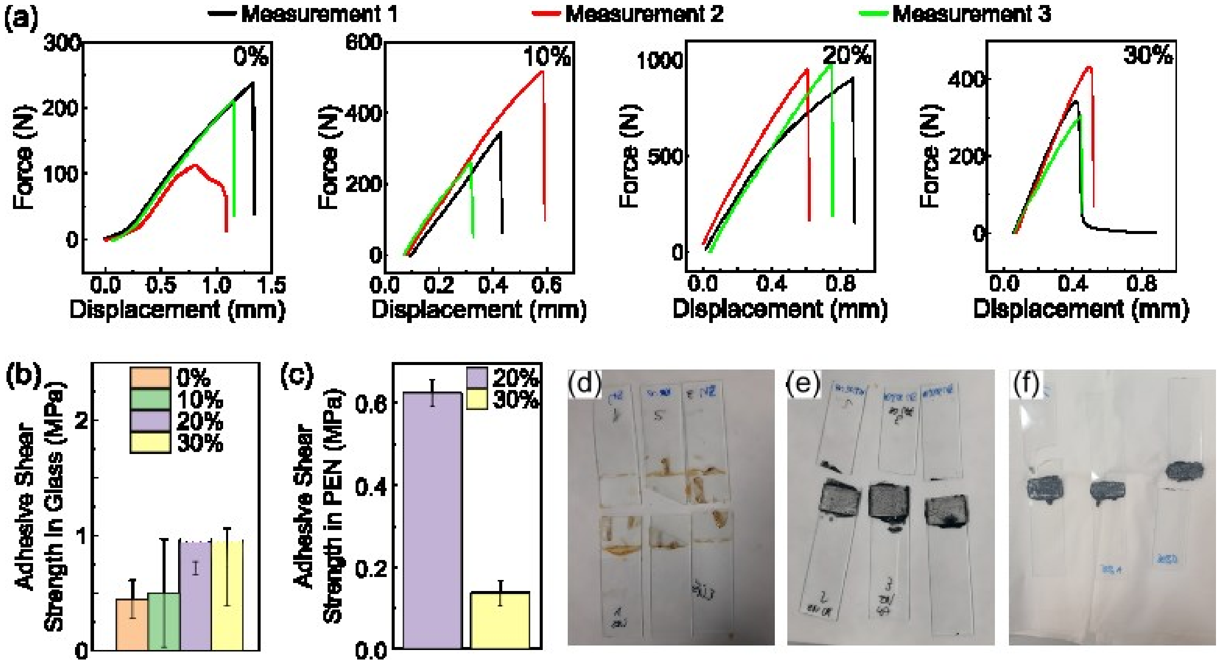

Figure S6. (a) Lap-shear displacement curves of 0%, 10%, 20% and 30% glues in aluminum substrates. (b) Adhesive shear strength of the edible glues on glass. Values for 20% and 30% are faded in to represent the expected higher values than 0.7 MPa due to substrate failure. (c) Adhesive shear strength of the 20% and 30% edible glues on PEN. (d) Photograph of the 0% on glass samples after lap shear experiment. (e) Photograph of the 20% on glass samples after lap shear experiment. (f) Photograph of the 30% on glass samples after lap shear experiment.

Good lap shear experiments could be carried out on aluminum obtaining highly reproducible displacement curves as the ones displayed in Figure S6a. Figure S6b and c show the adhesion lap shear strength of edible glues on glass and Polyethylene Naphthalene (PEN) respectively. For glass, we measured adhesion strength values of 0.45 ± 0.16 MPa in the case of pure zein, while, in the case of 20% and 30% AC content, fracture of glass (Figure S6d-f) occurs at 0.72 ± 0.06 MPa and 0.73 ± 0.33 respectively, before the detachment of the two substrates. The latter allows to assign an adhesion force in excess of 0.7 MPa to the 20% and 30% glue formulations. For PEN substrates, we measured adhesion strength values of 0.63 ± 0.03 MPa and 0.14 ± 0.03 MPa for 20% and 30% AC content respectively, confirming the 4x factor drop in the lap shear strength upon increasing the AC content as seen for aluminum.

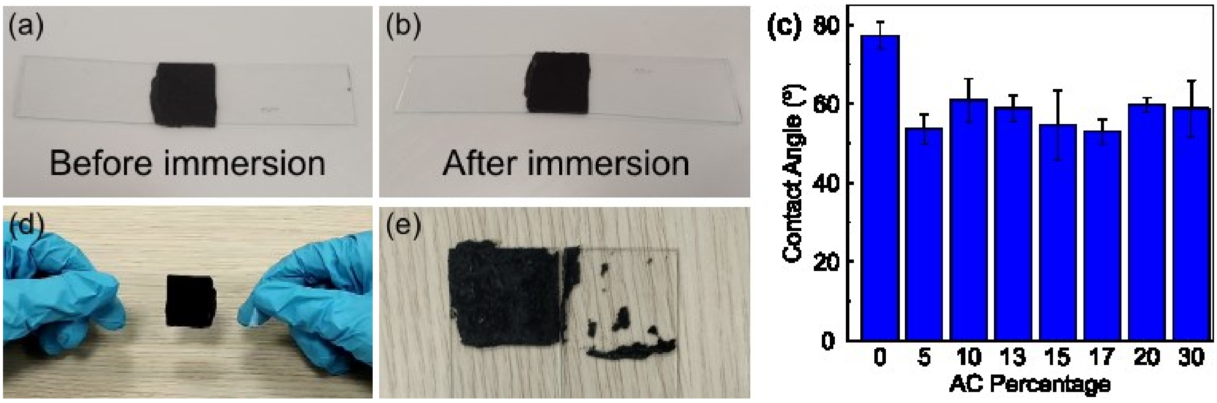

Figure S7. Photographs of glass slides glued with 20% AC glue (a) before and (b) after 24h under water. Adhesion remains over 24h under water despite glue swelling. (c) Water contact angle of the different formulations of glue. Photographs of the glued glass slides after 24h under water (d) before and (e) ungluing by hand.

As shown in Figure S7a and b, glass slides adhered between them with the edible conductive adhesive remain intact after immersion under water for 24h similar to the behavior reported for pure zein.[3] Notably, the incorporation of AC particles slightly reduces the water contact angle the edible glue from 77 ± 3º to values of around 55º-60º as shown in Figure S7c, rendering it thus slightly more hydrophilic. Despite this increased hydrophilicity, the adhesive maintains its adhesion under water. However, adhesion strength seems to decrease since the substrates can be easily separated by hand, as seen in Figures S7d and e. This effect has been already reported in other zein-based adhesives.[3]

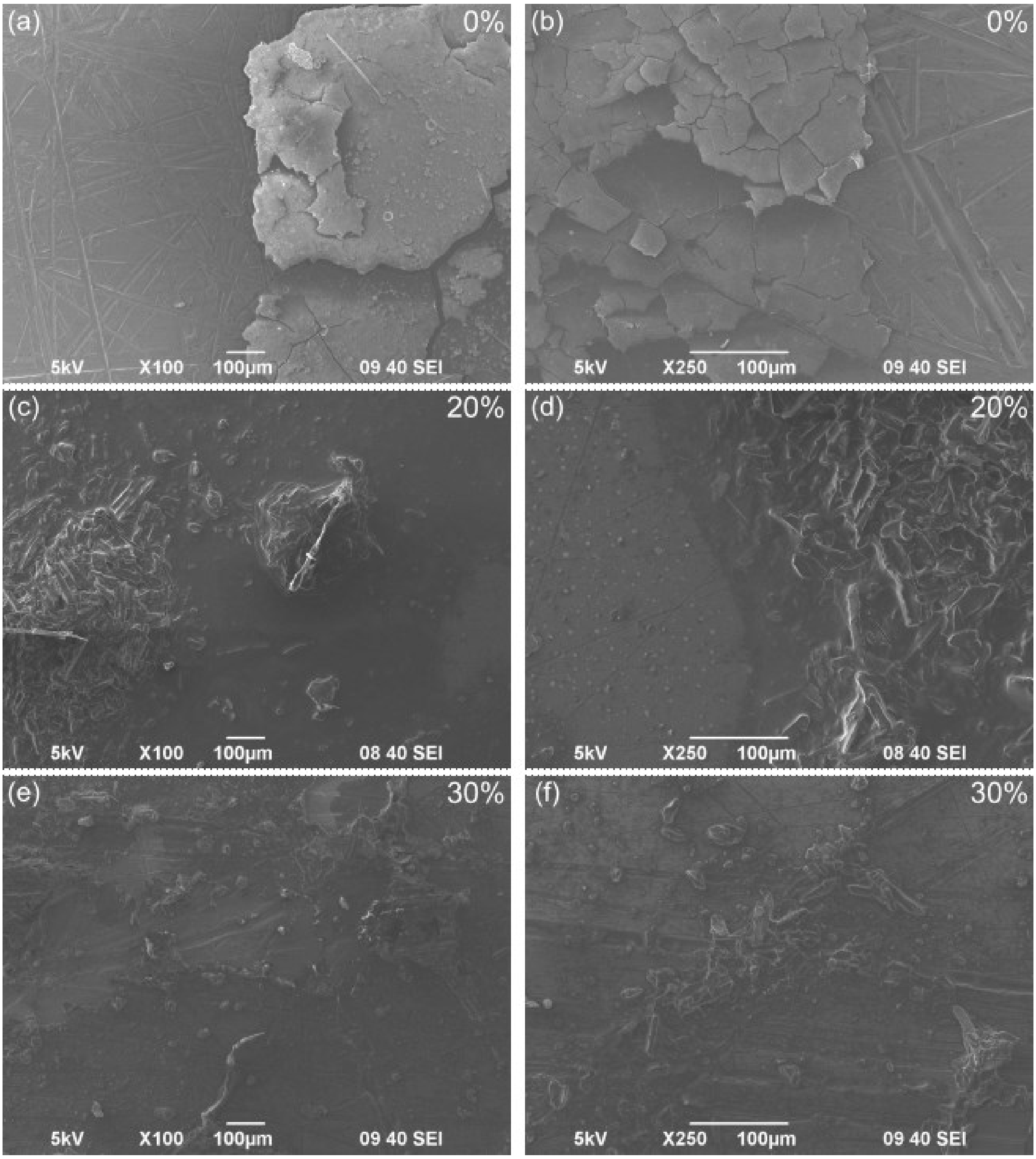

Figure S8. Top view SEM images of aluminum substrates after lap shear experiments having been previously glued with (a) and (b) 0% AC glue, (c) and (d) 20% AC glue and (e) and (f) 30% AC glue.

Interestingly, 0% glues after lap shear adhesion display large surface areas with fractured material as seen in Figure S8a and b. On the other hand, images of glue containing AC show homogeneous and robust blends of zein and AC, still after the lap shear experiments (Figure S8c-f). Consistently, lap shear experiments of the 0% are characterized by a cohesive failure of the material whilst 20% and 30% samples show an adhesive failure. Furthermore, the larger AC percentage in the 30% adhesive leads to lower coverage of AC particles with the adhesive zein and consequently to lower lap shear strength.

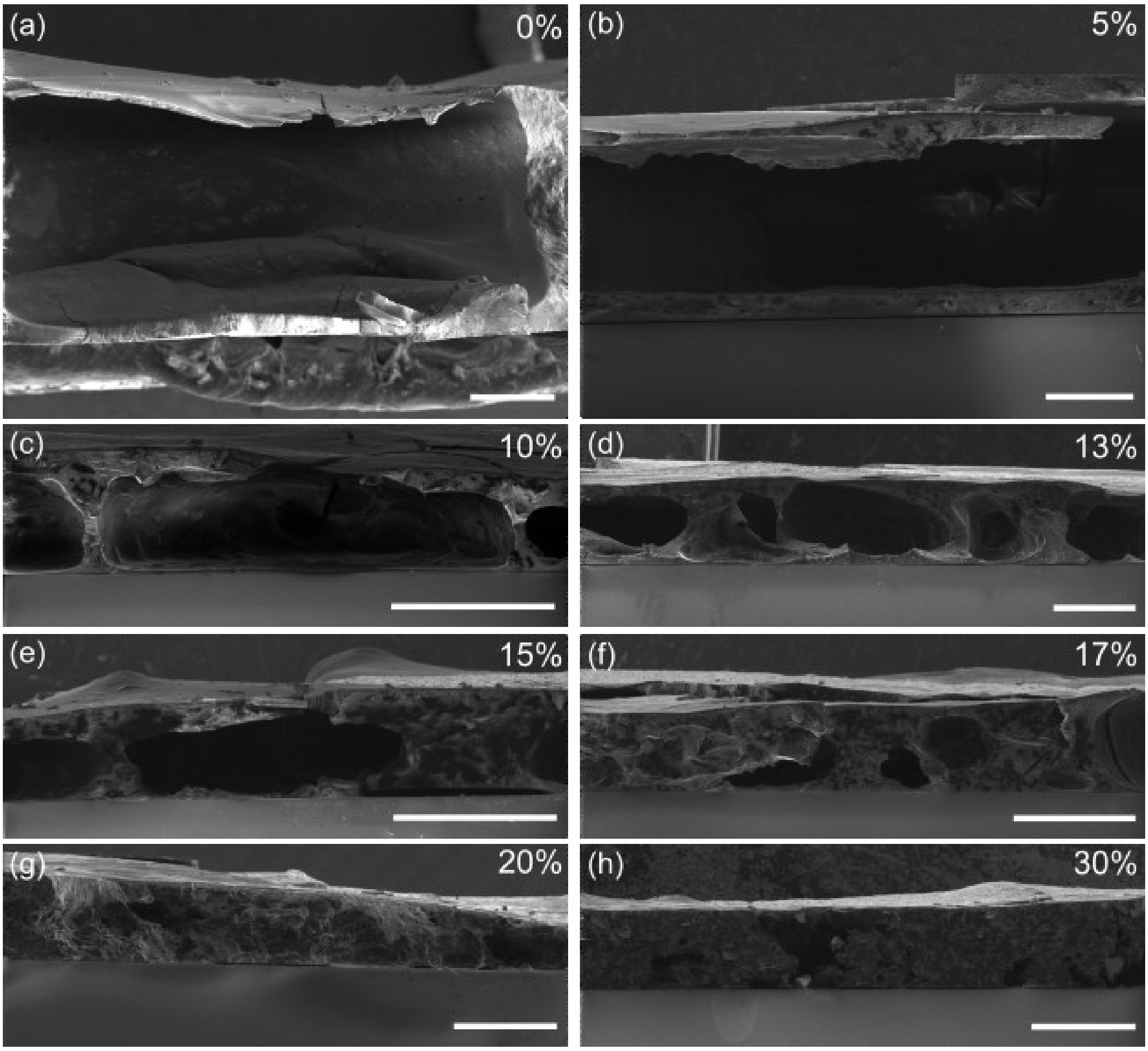

Figure S9. (a-h) Cross-section SEM images of brushed edible glues with different AC content after annealing at 80ºC. The AC content is indicated in each image. All scale bars are 200 µm.

Cross-section SEM images of the different glue formulations after single brush and annealing at 80ºC are displayed in Figure S9. Noteworthy, low AC content glues (from 0% to 13%) appear as a thick and smooth material but with large voids obtaining an unequal density of zein in the dried glue. Interestingly, upon increasing AC amount, voids within the dried material are diminished in size and density. On the other hand, 15% and 17% samples are more compact and thinner layers with discrete voids found along the dried glue. Finally, 20% and above AC loading dried glues present a high homogeneity and very smooth surfaces.

3. Electrical performance of the edible conductive glues

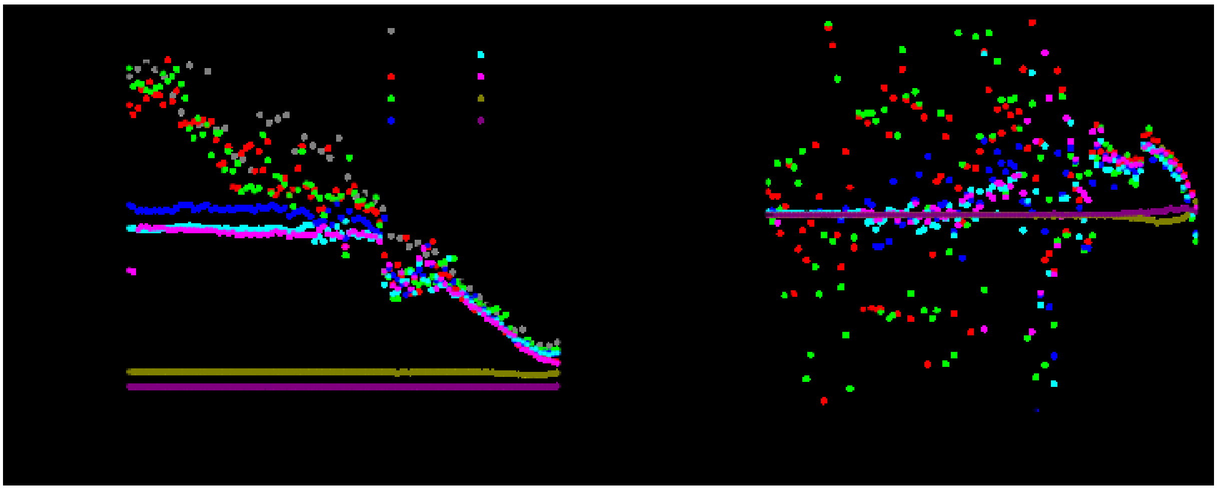

Figure S10. (a) Modulus and (b) phase of the impedance measurements for different glue formulations according to AC content

For glues from 0% to 10%, impedance modulus $|Z| > 10^{11}\ \Omega$ at 0.01 Hz and scattered phases corresponding to an open circuit can be observed in Figure S10a and S10b respectively. Upon increasing the AC content to a 13% -17% range, glues are characterized by a large $10^8 > |Z| > 10^7\ \Omega$ impedance with a near null phase at low frequencies which prompts to open circuit behavior above 10-100 Hz. Fully resistive behaviors at any frequency are seen only for 20% and 30% conductive glue formulations with impedance values $10^5 > |Z| > 10^4\ \Omega$.

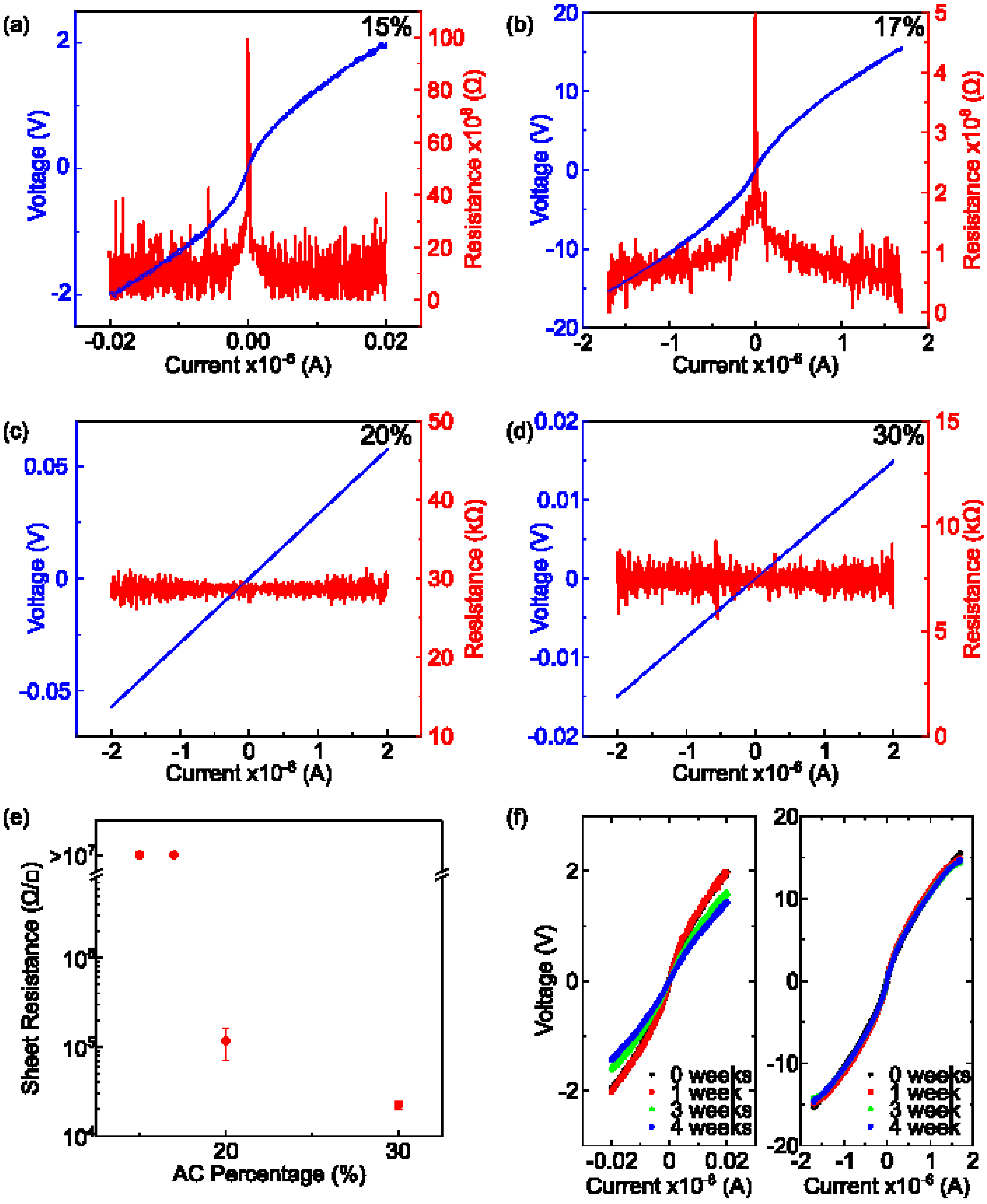

Figure S11. (a) Exemplary four-point resistance measurement of a 15% dried glue. (b) Exemplary four-point resistance measurement of a 17% dried glue. (c) Exemplary four-point resistance measurement of a 20% dried glue. (d) Exemplary four-point resistance measurement of a 30% dried glue. (e) Sheet resistance of edible conductive glues with different AC contents. (f) Four point measurement variation of the 15% and 17% glues over time.

For percentages from 0% to 13%, the resistance was above the limit of measurement of the equipment (1GΩ). For AC loadings of 15% and 17%, non-linear I-V curves were obtained (Figure S11a and b). Notably, a large decrease in the resistance is observed upon increasing the AC loading to 20% passing from non-linear to linear I-V curves confirming the formation of a full percolation path from this filler percentage loading onwards (Figure S11c and d). These measurements permitted the extraction of the sheet resistance of the 20% and 30% glues (Figure S11e). It is worth to note that contrarily to conductive glues, 15% and 17% measurements are not stable over time (Figure S11f).

4. Additional experiments on application of the edible conductive glue

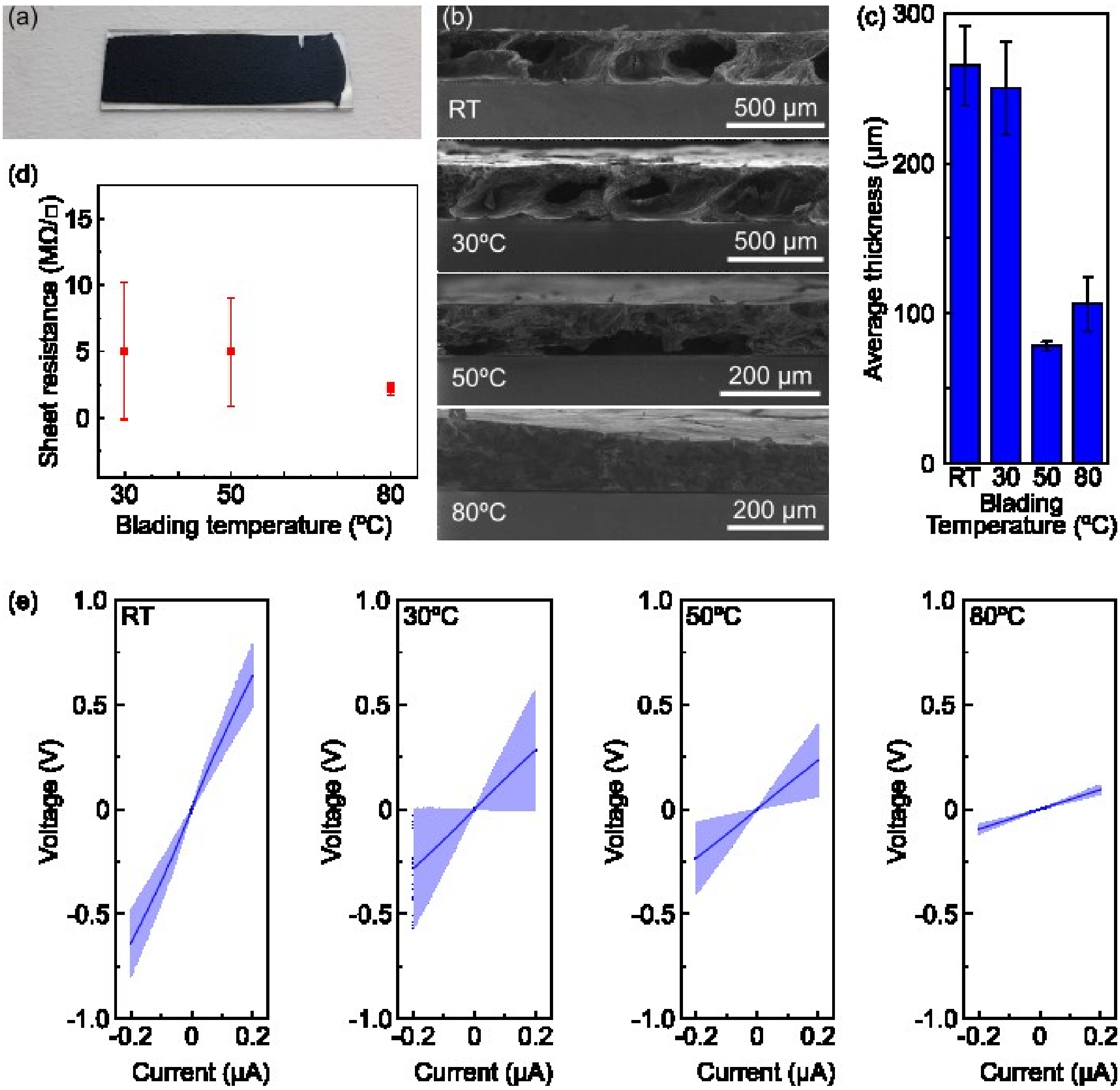

Figure S12. (a) Photograph of a bladed film of our edible conductive adhesive. (b) Cross-sectional images of films of 20% AC zein glue bladed at different temperatures. (c) Average thickness of the bladed films. (d) Sheet resistance of the different balding films depending on the bladed temperature. (e) Mean four-point I-V and mean resistance measurement of samples bladed at different temperatures. Exemplary variation of the resistance are shown in red.

Viscosities achieved at high temperatures have enabled compatibility of our edible conductive glues with blade-coating printing, leading of films as in Figure S12a blading on top of glass at 80 ºC at a constant shear rate of 5 mm/s. Depositions conducted at lower temperatures, where the ink viscosity exceeds the blade-coating compatibility limit (RT, 30 ºC, and 50 ºC), exhibit significant porosities, resulting in irregular material density within the films (Figure S12b). In contrast, films bladed at 80 ºC present as smooth and continuous materials. Attaining compatible viscosities allows for thinner depositions, as shown in Figure S12c measurements. The inconsistent material density and porosity in samples bladed at temperatures below 80 ºC lead to higher and more dispersed four-point sheet

resistance values (Figure S12d). Conversely, blading at 80 ºC produces more reproducible and lower sheet resistances, achieving resistivity values of 23 ± 7 kΩ·cm. Sheet resistances were extracted from the very linear I-V curves obtained from 30ºC onwards as seen in Figure S14b. The significant porosity in RT bladed samples resulted in variable I-V curves, with some samples exhibiting linear behavior and others non-linear. Therefore, viscosity modification via the temperature is essential not only for uniform deposition by blade-coating but also for optimizing the electrical properties of the films and the formation of a percolation path.

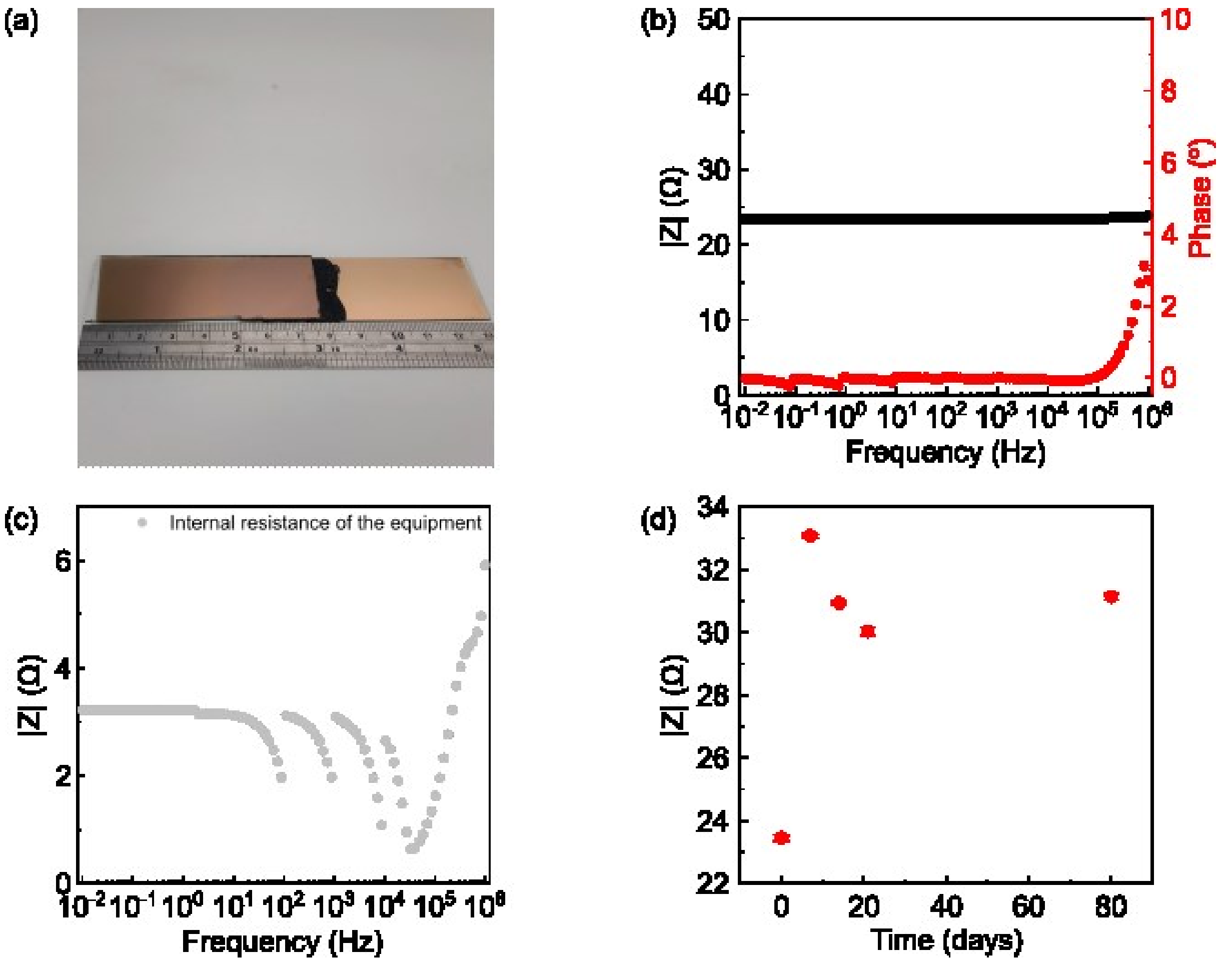

Figure S13. (a) Photograph of gold-coated glasses glued with the 20% edible conductive glue conforming a vertical interconnection. (b) Electrical impedance spectroscopy (EIS) measurement of the vertical interconnection. (c) Internal resistance of the EIS analyzer. (d) Evolution of the EIS measurement of the vertical interconnection over time.

The 20% edible glue was deposited on top of gold-coated glass substrates which were further adhered together to simulate the vertical interconnection between devices (Figure S13). Noteworthy, adhesion on gold coated substrates exhibited similar mechanical stability comparable to that observed on previously used substrates. Electrical impedance spectroscopy between the metallic surfaces indicated a very low impedance of ≈ 23 Ω are obtained with null phase, displaying a good ohmic contact and without introducing undesired capacitive effects. Consequently, the 20% demonstrates excellent electrical performance, providing low resistive loads in vertical interconnections. Furthermore, the impedance measurements remained consistent over several months, indicating substantial adhesive and electrical stability over time.

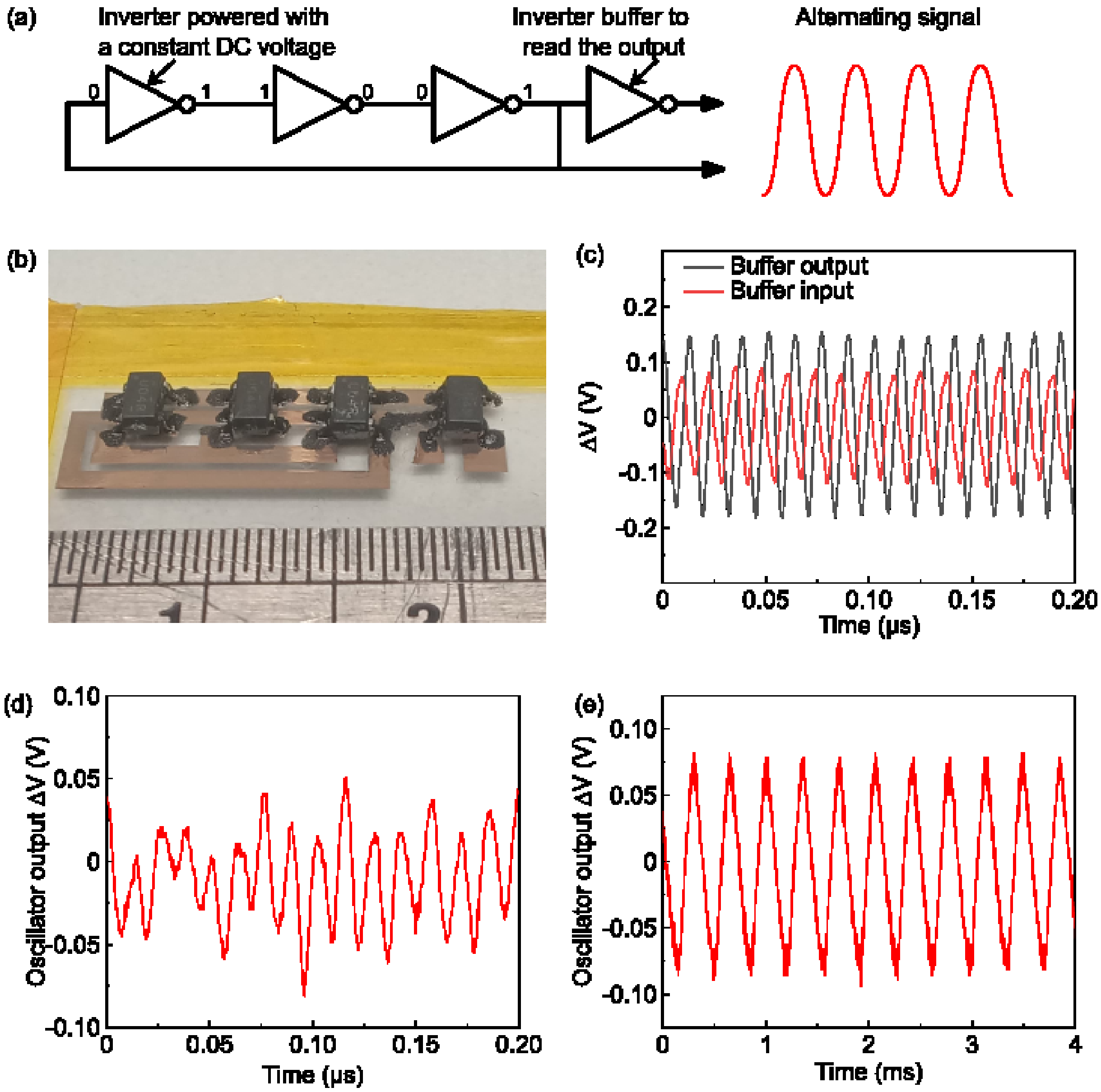

Figure S14. (a) Schematic of the implemented ring oscillator and expected signal. (b) Surface-mounted ring oscillator using commercial SN74AUC1G04 inverters mounted onto an ink-jet printed gold circuit on edible ethyl cellulose substrate fitted into a 00 commercial gelatin capsule. (c) Read voltages at the output buffer inverter and before the buffer inverter, i.e. in the ring oscillator when providing 1.2V to the oscillator. (d) Read voltage at the output buffer inverter when providing 800mV to the oscillator. (e) Read voltage at the output buffer inverter when providing 300 mV to the oscillator.

In a ring oscillator circuit, the odd number of inverters creates an alternation between 0 and 1 logic states, generating an alternating signal within the ring (Figure S14a). The alternating signal can be read at the ring itself or at an inverter used as an output buffer reader. The final surface-mounted ring oscillator is shown in Figure S14b, following the same three inverter structure with a fourth as output reader. The circuit's performance was validated by comparing the oscillating signals within the ring (prior to the buffer) and after the output buffer (Figure S14c). Notably, the signals are inverted relative to each other, the maxima before the buffer correspond to the minima after the buffer, both maintaining

a frequency of 78 MHz. This demonstrates perfect inversion by the output buffer and indicates a robust connection using our edible glue. A 74 MHz albeit distorted oscillation was observed when providing 800mV, the minimum supply voltage of the commercial inverters, further confirming the proper assembly of the circuit with the glue (Figure S14d). The absence of a clean oscillation may be attributed to the resistance of the overall circuit. When applying 300 mV, below the minimum recommended voltage, an oscillation with a much lower frequency of 2.8 kHz was observed (Figure S14e).

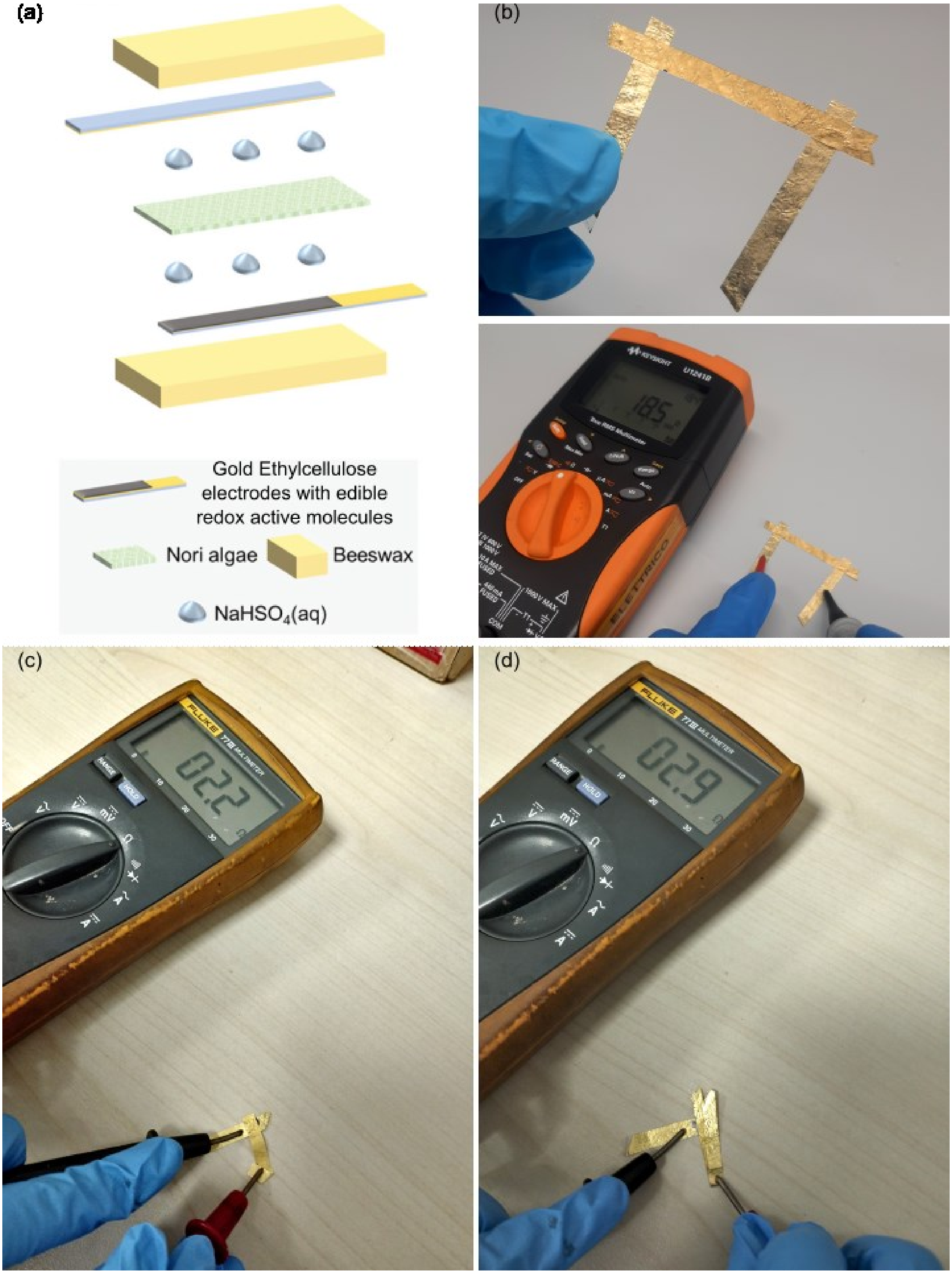

Figure S15. (a) Scheme of the battery adapted from [4]. (b) Glued electrodes lifted to show large adhesion and mechanical stability of the interconnection. Photograph of a resistance measurement of glued edible electrodes made with ethyl cellulose and gold foil. Resistance measurement between ethyl cellulose electrodes interconnected with (c) our edible glue and (d) commercial silver epoxy.

Fully edible rechargeable batteries are built by using only food-grade materials as redox-active vitamin (riboflavin) and edible dye (quercetin) deposited on gold edible laminated on ethyl cellulose as current collectors, a nori algae soaked in a sodium hydrogen sulfate as separator and a beeswax encapsulation layer (Figure 15a). Thus, electrodes as those used in the edible batteries (made from ethyl cellulose films with edible gold leaves laminated on top) were pasted via ink brushing. A small amount of glue (one brush) was required for adhesion of edible electrodes resulting in a fast drying of the glue at RT (less than 30 min). The adhesion force of the glue on ethyl cellulose is enough to allow manipulating and maintain a self-standing structure as seen in Figure S15b. The measured electrical resistance from the first to the last electrode presents very low values of 18.5 Ω, suggesting good ohmic contact. Indeed, interconnections between laminated gold ethyl cellulose electrodes achieved with our material provided low resistances of 2.2 Ω, similarly to the 2.9 Ω obtained with commercial non-edible conductive epoxies (Figure S15c and d).

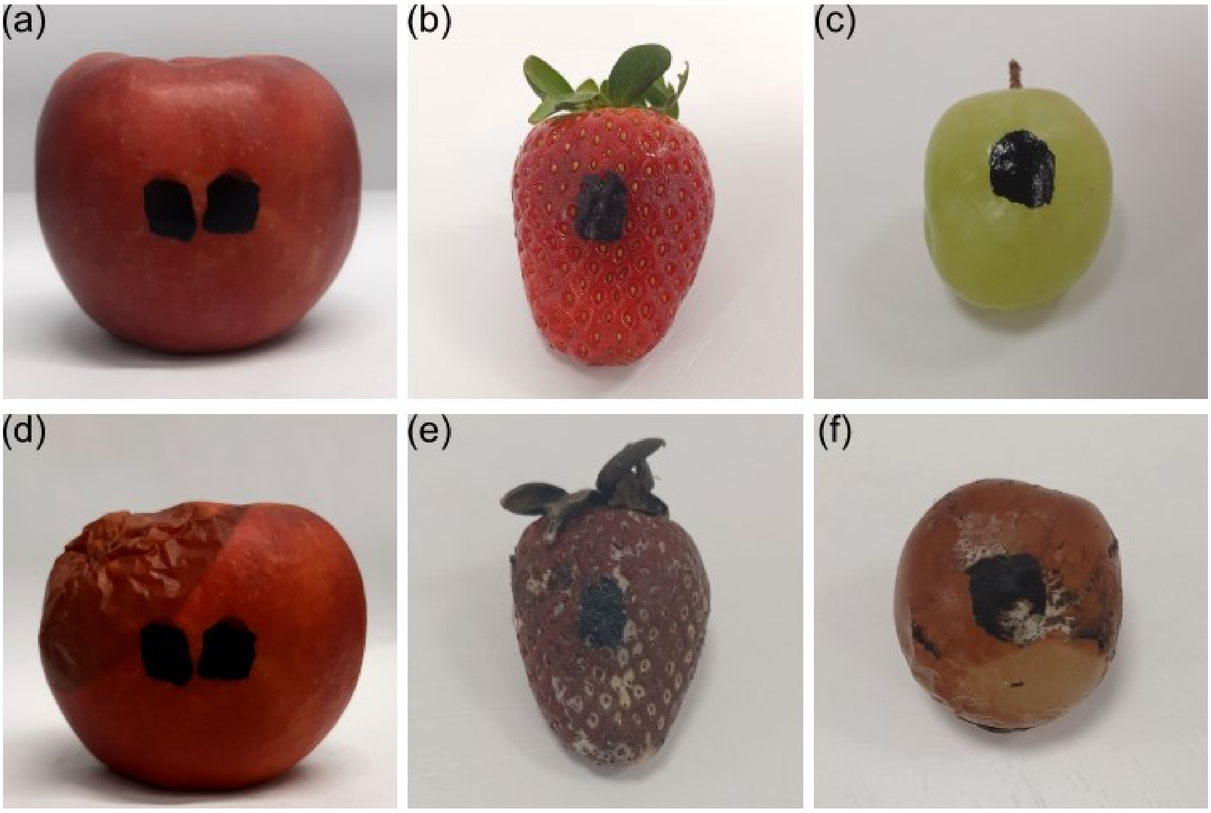

Figure S16. Fresh (a) apple, (b) strawberry and (c) grape with painted electrodes made with edible conductive glue. Same fruits after rotting: (d) apple, (e) strawberry and (f) grape.

Edible electrodes for electrical bio-impedance spectroscopy were achieved by depositing a light coating of our edible conductive glue on top of different fruits via ink brushing as seen in Figure S16a-c. The dried electrodes exhibit excellent conformability to the diverse surfaces of the fruits. Notably, the electrodes on apples and grapes maintain the curvature of the fruit without cracking, even after drying. Additionally, the complex indentations in the strawberry skin, caused by its seeds, are accurately replicated by the edible electrodes. Remarkably, electrodes remain on the fruit skin over long periods (one month for apple, two weeks for strawberry and grape) without problems or detaching despite natural changes in the fruit as seen in Figure S16d-f.

4. Supplementary Videos description

A total of six supplementary videos have been submitted alongside the paper. Videos S1-S3 demonstrate the easy deposition of the edible conductive glues via ink brushing permitting tailored shapes. Video S1 shows the deposition of a connecting line, Video S2 shows the writing of group and institutional acronyms and Video S3 the deposition of a curved line. The video speed for these three videos has been accelerated (x2). Video S4 demonstrates the compatibility of the edible conductive inks with masking. Two tapes have been applied to the substrate with a narrow 1 mm wide gap between them. After application of the ink, the tapes are immediately removed providing a 1 mm wide line of the edible conductive glue. The video speed has been gradually accelerated (from x1 to x2 when removing the tape). Video S5 is a collage of videos of the visual viscosity and the compatibility with brushing of the different edible glues depending on the AC content. As shown, inks up to 30% AC display a large liquid behavior compatible with ink brushing as only a light amount of ink is taken by the brush. For 40%, a large viscous behavior is observed. For 50% a large resin like behavior is obtained. For both of these inks, the brush takes a large ink volume which would lead to uncontrolled depositions. Video S6 shows the good adhesion of the edible conductive glues between common laboratory objects as a glass slide and a glove.

5. References of the Supporting Information

[1] A. Messaâdi, N. Dhouibi, H. Hamda, F. B. M. Belgacem, Y. H. Adbelkader, N. Ouerfelli, A. H. Hamzaoui, *J Chem* **2015**, *2015*, 1.
[2] J. W. Lawton, *Cereal Chem* **2004**, *81*, 1.
[3] Y. Wei, J. Yao, Z. Shao, X. Chen, *ACS Sustain Chem Eng* **2020**, *8*, 7668.
[4] I. K. Ilic, V. Galli, L. Lamanna, P. Cataldi, L. Pasquale, V. F. Annese, A. Athanassiou, M. Caironi, *Advanced Materials* **2023**, *35*, DOI 10.1002/adma.202211400.
